# COGNITIVE IMPAIRMENT IS ASSOCIATED WITH INFERIOR POSTURAL BALANCE AND REDUCED GAIT SPEED

**DOI:** 10.1093/geroni/igad104.2941

**Published:** 2023-12-21

**Authors:** Laura Katharina Schmidt, Tim Stuckenschneider, Jessica Koschate, Tania Zieschang

**Affiliations:** Carl von Ossietzky University Oldenburg, Oldenburg, Niedersachsen, Germany; Carl von Ossietzky University, Oldenburg, Nordrhein-Westfalen, Germany; Carl von Ossietzky University, Oldenburg, Niedersachsen, Germany; Carl von Ossietzky University, Oldenburg, Niedersachsen, Germany

## Abstract

Fall-related sequelae including balance as well as gait impairments are more pronounced in older adults with cognitive impairment (CIOA) compared to cognitively healthy older adults (CHOA). Evidence is scarce about differences in postural balance and gait in CHOA and CIOA after a fall, even though these are major risks for recurrent falls. Especially, there is a dearth of data based on validated sensor technologies. Thus, it is our aim to use sensor-based data to investigate differences between these two groups. Postural balance and gait were measured using three wirelessly synchronized IMU’s (APDM, Inc., Portland, USA) in n=50 CHOA, mean age 73.82±8.25 years and n= 56 CIOA, mean age 78.27±8.0 years. Data were collected at participants’ homes as part of a comprehensive geriatric assessment in the “SeFallED” study within four weeks after presentation to the emergency department (ED) after a sentinel fall (German Clinical Trials Register ID: 00025949). Differences in postural balance and gait between two groups were analyzed by using a Mann-Whitney-U-Test. Statistical analysis revealed a significantly poorer postural balance in CIOA compared to CHOA for the parameters Jerk (m2/s5; p=0.039) and mean velocity (m/s; p=0.036). Further, CIOA had a significantly lower gait speed (m/s; p=0.035) and stride length (m/s; p=0.028) compared to CHOA. Even though both groups had shortly experienced a fall with presentation to the ED, impairments in gait and balance parameters were more prominent in CIOA. Based on these results, physical exercise interventions should focus on the improvement of impaired gait speed and balance particularly in CIOA.

